# Comprehensive Analysis of the SUV Gene Family in Allopolyploid *Brassica napus* and Its Diploid Ancestors

**DOI:** 10.3390/genes12121848

**Published:** 2021-11-23

**Authors:** Meimei Hu, Mengdi Li, Jianbo Wang

**Affiliations:** 1 State Key Laboratory of Hybrid Rice, College of Life Sciences, Wuhan University, Wuhan 430072, China; meimeihu@whu.edu.cn (M.H.); mengdili@whu.edu.cn (M.L.); 2 Key Laboratory of Resource Biology and Biotechnology in Western China, Ministry of Education, College of Life Sciences, Northwest University, Xi’an 710069, China

**Keywords:** *Brassica*, SUV gene family, evolutionary relationship, gene expansion, gene loss, gene expression

## Abstract

SUV (the Suppressor of variegation [Su(var)] homologs and related) gene family is a subgroup of the SET gene family. According to the SRA domain and WIYLD domain distributions, it can be divided into two categories, namely SUVH (the Suppressor of variegation [Su(var)] homologs) and SUVR (the Suppressor of variegation [Su(var)] related). In this study, 139 SUV genes were identified in allopolyploid *Brassica napus* and its diploid ancestors, and their evolutionary relationships, protein properties, gene structures, motif distributions, transposable elements, *cis*-acting elements and gene expression patterns were analyzed. Our results showed that the SUV gene family of *B. napus* was amplified during allopolyploidization, in which the segmental duplication and TRD played critical roles. After the separation of *Brassica* and Arabidopsis lineages, orthologous gene analysis showed that many SUV genes were lost during the evolutionary process in *B. rapa*, *B. oleracea* and *B. napus*. The analysis of the gene and protein structures and expression patterns of 30 orthologous gene pairs which may have evolutionary relationships showed that most of them were conserved in gene structures and protein motifs, but only four gene pairs had the same expression patterns.

## 1. Introduction

The suppressor of variegation [Su(var)] homologs and related (SUV) gene family is a general term for these genes coding SET (Suppressor of variegation collection, Enhancer of zeste, Trithorax) domain proteins. SET gene family was first found in *Drosophila* and divided into four subgroups [1]. The SET domain (named by three proteins in *Drosophila*, namely Suppressor of variegation collection, Enhancer of zeste and Trithorax) has histone methyltransferase activity [2,3,4]. The 37 putative SET domain genes in Arabidopsis were divided into four categories: Enhancer of zeste [E(z)] homologs, Ash1 homologs and related, trixthorax(trx) homologs and related and Suppressor of variegation [Su(var)] homologs and related [5]. The SET domain gene families in Arabidopsis and maize were divided into five categories by their genetic relationships [6]. According to the combination of SET domain with different motifs or domains (such as PHD, PWWP, MBD, AWS, Pre-SET and SRA), the SET gene family can be divided into different categories, and the conservation of these domains in different subgroups show that they play an important role in their respective subgroups. SRA domain and WIYLD domain are only found in plant SET proteins [7]. SUV gene family can be divided into SUVH (Suppressor of variegation [Su(var)] homologs) and SUVR (Suppressor of variegation [Su(var)] related) genes by the existence of SRA domain, and the former contains SRA domain, while the latter does not [8].

SET domain genes of the SUV gene family are characterized by the Pre-SET-SET-Post-SET model, and the acquisition of some other conserved domains which work together with the SET domain play complementary roles [5]. There are 10 SUVH genes and 5 SUVR genes in the SUV gene family in Arabidopsis, and the Post-SET domain of some members has been lost during evolution [2,9]. In addition, the evolution of the SET gene family found that SRA was only found in plants, and *SUVH7* and *SUVH10* were probably pseudogenes [7]. Gene silencing in eukaryotes is related to the formation of heterochromatin. Su(var)3-9 protein, the domain modifier of heterochromatin-induced gene silencing plays an important role in the correct assembly of heterochromatin [10,11]. Studies have found that [1,12], Su(var)3-9 gene, which belongs to the haplo suppressor locus group with triple enhancer effect, mediates the expression of heterochromatin-related genes, and shows to be dose-dependent. SUVH not only contains the Pre-SET, SET and Post-SET, but also contains the SRA domain. In Arabidopsis, SRA domain (RING finger related domain) proteins play a key role in three main DNA methylation pathways controlled by MET1, CMT3 and DRM2 methyltransferase [5,13]. The SUVH1, SUVH4, SUVH5 and SUVH6 proteins were proved to be H3K9 methyltransferases [3]. Different from SUVH, SUVR lacks the SRA domain, but a new WIYLD plant-specific domain has been identified in SUVR1, SUVR2 and SUVR4 [4]. Whole-genome duplication is one of the reasons for gene family amplification. During the evolution process of the SET gene family, many gene replication events occurred, and segmental duplication was the main reason for the amplification of the SET gene family (especially for the SUV subfamily) [7,14]. 

At present, the SET gene family has been analyzed in *B. rapa* [15,16], rice [17], bread wheat [18] and apple [19], etc. With the development of the research on the SET gene family, the role of the Su(var) 3-9 SET gene (SUV gene) family in DNA methylation is gradually revealed. The SUV gene family in Arabidopsis can be divided into seven categories, among which V-1, V-2, V-3, V-5 are SUVHs, and V-4, V-6, V-7 are SUVRs. The phylogenetic relationships indicate that some additional domains, such as SRA, ZnF-C2H2 and WIYLD, may be integrated into genes very early [8]. In Arabidopsis, SUVH2 plays an important role in histone methylation and gene silencing [20]. SUVH2 and SUVH9 do not directly catalyze DNA methylation but regulate gene expression by participating in the RdDM pathway [21,22]. The structures of SUVH5 and SUVH6 and the modes of action involved in methylation regulation were revealed [23,24,25,26]. In addition, SUVH1 can regulate gene expression by participating in promoter methylation and has been confirmed to be closely related to LUC gene expression [27]. The WYILD domain in SUVRs regulates methylation by binding ubiquitin [28]. In conclusion, two categories of the SUV gene family (SUVH and SUVR) are significant for regulating DNA methylation and gene expression in different ways. The SUVH is related to the maintenance and regulation of heterochromatin structure through its SRA domain participating in the RdDM process, while the SUVR mainly plays a role by WYILD domain combining with ubiquitination H2B [29].

The *Brassica* is a genus of Brassicaceae, and some of the species in this genus are widely grown as vegetables and oil crops, which brought huge economic value for the development of human beings. The U’s triangle was used to describe several representative species of *Brassica* which mainly included three diploid species (*B. rapa*, *B.* *nigra* and *B. oleracea*) and three allotetraploid species (*B. juncea*, *B. napus* and *B. carinata*) and the allotetraploid species were produced by hybridizing diploids and polyploidization [30]. Allopolyploidization has led to dramatic changes in the allopolyploid genome, finally, the genome appears the phenomenon of asymmetric evolution and expression after the long and complex selection process [31]. Polyploidization is a common phenomenon in the plants. After the separation of *Brassica* and Arabidopsis lineages, there was a whole-genome triplication (WGT) event, followed by a diploidization process that resulted in a large loss of *Brassica* genome. Compared with the genome of Arabidopsis, the diploidized genome of *B. rapa* was greatly reduced and gene loss was common [32]. After genome diploidization, the scale of the gene family was changed. The gene family changes of the genome may contribute to the morphological differentiation of different species in *Brassica* [33]. The glucosinolate genes were lost after the formation of *B. napus*, but the oil biosynthesis genes were amplified [34].

SET gene family has been reported in many species. Accordingly, due to the critical role of the Su(var) 3-9 SET gene in DNA methylation, the SUV gene has gradually occupied the attention of researchers. So far, except for *B. rapa*, the research of the SUV gene family in *B. oleracea* and *B. napus* is still blank. In this study, we identified the SUV gene family in *B. napus* and its diploid ancestral species, and analyzed their phylogeny and revealed the evolutionary relationships and characteristics of the SUV gene family in *B. napus* and its diploid ancestors.

## 2. Materials and Methods

### 2.1. Materials and Transcriptome Sequencing

The seeds of sequencing materials, *B. napus* (cv. Darmor), *B. rapa* (cv. Chiifu) and *B. oleracea* (cv. Jinzaosheng) were from the Oil Crops Research Institute, Chinese Academy of Agricultural Sciences. The seeds were naturally planted in soil and bagged when flowering to avoid pollen pollution. The data for expression patterns analysis in this study came from the previous sequencing data of our research group [35]. The stems, leaves, flowers, siliques from *B. napus* (cv. Darmor), *B. rapa* (cv. Chiifu) and *B. oleracea* (cv. Jinzaosheng) were used for Illumina (HiSeq X-Ten).

### 2.2. Identification of SUV Gene Family

The Arabidopsis SUV proteins (10 SUVH proteins and 5 SUVR proteins) were obtained from the TAIR database (http://www.arabidopsis.org/, accessed on 20 April 2021). They were then were submitted to the BRAD database (http://39.100.233.196/#/, accessed on 20 April 2021) to BLASTp (E value < 1 × 10^−5^) [36]. Then, the syntenic genes of the SUV gene family in Arabidopsis were retrieved from the BRAD database to be a supplement of BLASTp [37]. The obtained ID was de-duplicated and the relevant protein sequences were extracted for domain retrieval. Three methods were used to screen the domains: the obtained protein sequences were submitted to CDD database (https://www.ncbi.nlm.nih.gov/, accessed on 15 March 2021) [38], SMART database (http://smart.embl-heidelberg.de/, accessed on 15 March 2021) [39] and Pfam database (http://pfam.xfam.org/, accessed on 15 March 2021) [40] for domain searching. SUV gene family belongs to a category of the SET gene family, the sequences of SET domain retrieved from all three databases will be retained as the final result and renamed by *Brassica* standard nomenclature [41]. The same methods were used to identify the SUV gene family in rice by using genome data (version 7.0) from the Rice Genome Annotation Project database (http://rice.plantbiology.msu.edu/, accessed on 15 March 2021) [42] and BLASTp in SNP-Seek database (https://snp-seek.irri.org/, accessed on 15 March 2021) [43]. 

### 2.3. Phylogenetic Analysis

At present, there are few studies on SUV gene family. In this study, we identified the SUV gene family of rice in the same way as the identification of *Brassica* SUV gene family. Finally, 26 SUV genes were identified in rice. The 26 SUV genes in rice and 15 SUV genes in Arabidopsis were used as outgroups of the *Brassica* SUV gene family to construct the phylogenetic tree. The fast tree service of NGphylogeny.fr (https://ngphylogeny.fr/, accessed on 20 April 2021) was used to infer an approximately-maximum-likelihood phylogenetic tree [44], which was prettified by the Interactive Tree Of Life (iTOL) (https://itol.embl.de/, accessed on 20 April 2021) [45].

### 2.4. Chromosomal Mapping and Gene Structure

The MapChart [46] was used to plot the gene chromosomal mapping situation. Gene structure annotation information of the SUV gene family was then extracted from GFF3 files. The gene structures were displayed by GSDS 2.0 (http://gsds.gao-lab.org/, accessed on 20 April 2021) [47] and the phylogenetic tree was constructed by the fast tree service of NGphylogeny.fr.

### 2.5. Protein Properties Prediction and Conserved Motif Analysis

The Expasy ProtParam Tool (https://web.expasy.org/protparam/, accessed on 20 April 2021) [48] was used to search the physicochemical characterization of SUV proteins, including the number of amino acids, molecular weight, grand average of hydropathicity, instability index and theoretical pI. The information of subcellular localization was predicted by WoLF PSORT (https://wolfpsort.hgc.jp/, accessed on 20 April 2021) [49]. To analyze the conserved status of the proteins encoded by the SUV gene family during evolution, we predicted the conserved motifs by NCBI CDD database (https://www.ncbi.nlm.nih.gov/, accessed on 15 March 2021). The MEME website was used to analyze the motif situation (http://meme-suite.org/tools/meme, accessed on 15 March 2021) [50].

### 2.6. The Syntenic Genes, Duplication Types and Transposable Elements Analysis

Fifteen AtSUV genes were used to search syntenic genes in the BRAD database (http://39.100.233.196/#/, accessed on 15 March 2021) [36], and the TBtools [51] was used to plot the syntenic relationships between them. The analysis of duplicate genes was divided into two steps. Firstly, the sequences of SUV genes were submitted to NCBI to BLASTp (https://blast.ncbi.nlm.nih.gov/, accessed on 15 March 2021). Then, the genes whose coverage and identity were greater than or equal to 80% were retained and combined into duplicate gene pairs (the pairs can only be duplicated if two genes are BLASTp bidirectional). The values of Ka, Ks and Ka/Ks of the duplicated gene pairs were calculated by TBtools, and the selection types during evolution were analyzed. The 2000 bp upstream and downstream sequences were extracted and submitted to the Repeat Masking tools (https://www.girinst.org/censor/index.php, accessed on 15 March 2021) of REPBASE database [52] to predict the TEs around the SUV gene locus in *Brassica*. 

### 2.7. Cis-Elements and SUV Genes Expression

To analyze *cis*-elements, the sequences upstream 1500 bp of SUV genes were extracted to submit to the PlantCARE website (http://bioinformatics.psb.ugent.be/, accessed on 15 March 2021) [53], and finally, the data was used to display the results of the *cis*-elements. The transcriptome data (SRR7816633-SRR7816668) was used to analyze the expression mode of the SUV gene family. The FPKM (Fragments Per Kilobase of transcript per Million mapped reads) value calculated by RSEM (Expectation-Maximization) tool was to be a standard of gene expression level [54], and then, results were plotted into a heatmap by TBtools. 

## 3. Results

### 3.1. Identification, Characterization of SUV Gene Family and the Protein Properties Prediction

The protein sequences of SUV family in Arabidopsis were submitted to the BRAD database [36] to BLASTp (E value < 1 × 10^−5^), and the database versions were Bra1.5, Bol1.1 and Bna4.1. After removal of redundancy, 44, 41 and 99 SUV proteins in *B. rapa*, *B. oleracea* and *B. napus* were obtained, respectively. The obtained sequences were successively submitted to CDD, SMART and Pfam databases for domain filtering (filtering criteria: sequences containing SET domains were retained, while sequences lacking SET domains were discarded). Finally, the corresponding genes encoding SUV protein were obtained, including 35 genes in *B. rapa*, 29 genes in *B. oleracea* and 75 genes in *B. napus* (Table S1), with a total of 139 genes.

In Arabidopsis, the SUV gene family is mainly divided into two categories, namely AtSUVH and AtSUVR. The identified 139 SUV genes were renamed according to the SUV gene family in Arabidopsis and *Brassica* standard nomenclature. The “a” in the gene name indicates that the gene has the highest homology with the homologous gene of Arabidopsis, followed by “b”, and so on. The letters “A” and “C” in the name of the SUV gene in *B. napus* indicate that the gene is located in the A_n_ or C_n_ subgenome, respectively.

According to the data in Table S1, no homologous genes of *AtSUVR1*, *AtSUVH8* and *AtSUVH10* were identified in *B.* *rapa*, *B.* *oleracea* and *B.* *napus*. It can be speculated that after the phylogenetic separation of *Brassica* ancestors and Arabidopsis, these three orthologous genes of Arabidopsis have been lost. By analyzing the data in Table S1, it was found that the SUV genes in *B. napus* and *B. rapa*, which had the homology relationships with *AtSUVH2*, *AtSUVH4*, *AtSUVH6*, *AtSUVH9*, *AtSUVR2* and *AtSUVR4*, had equal numbers, and the number of genes of BnA.SUV and BrSUV were also corresponding to each other. However, for these homology genes of *AtSUVH1*, *AtSUVH3*, *AtSUVH7*, *AtSUVR3* and *AtSUVR5,* the number of SUV genes in *B. napus* was significantly higher than that in *B. rapa* and *B. oleracea*, indicating that the SUV gene family expansion occurred in the process of allopolyploidy in *B. napus*.

The Expasy ProtParam Tool and WoLF PSORT were used to predict the SUV protein characteristics and location. All SUV proteins are hydrophilic proteins (Table S2). The Instability indexes of 119 (86%) SUV proteins are greater than 40, indicating that the structures of most BnSUV proteins are unstable. The subcellular localization analysis of all SUV proteins by WoLF PSORT showed that all the 75 BnSUV proteins were predicted in the nucleus except for BnC.SUVR5d and BnC. SUVR4a. All the 35 BrSUV proteins except for BrSUVR5c and BrSUVR4b were predicted in the nucleus. There were 58 SUV genes in *B. napus* predicted in the nucleus, 9 in the cytoplasm, 6 in the chloroplast, 1 in the lysosome and 1 in the cell membrane, respectively. In *B.* *rapa*, 27 SUV proteins were predicted to be located in the nucleus, 3 in the chloroplast, 3 in the cytoplasm and 1 in the cell membrane. There were 19, 6, 3 and 1 SUV proteins of *B. oleracea* in nucleus, cytoplasm, chloroplast and cell membrane, respectively. The predicted location information of most BnSUV proteins is the same as that of *B. rapa* and *B. oleracea*, and they are all localized in the nucleus, chloroplast and cytoplasm. Location analysis of SUV proteins predicted in nucleus, chloroplast and cytoplasm found that approximately 75% (104) of SUV proteins localized in nucleus. Proteins located in chloroplast are mainly SUVR5, SUVH2, SUVH5 and SUVH7, while proteins localized in cytoplasm are mainly SUVR2, SUVR4, SUVR5 and SUVH2. The localization differences of proteins encoded by SUV gene family might be related to their functional differentiation.

### 3.2. The Phylogenetic Relationship Analysis of SUV Proteins

**Figure 1:**
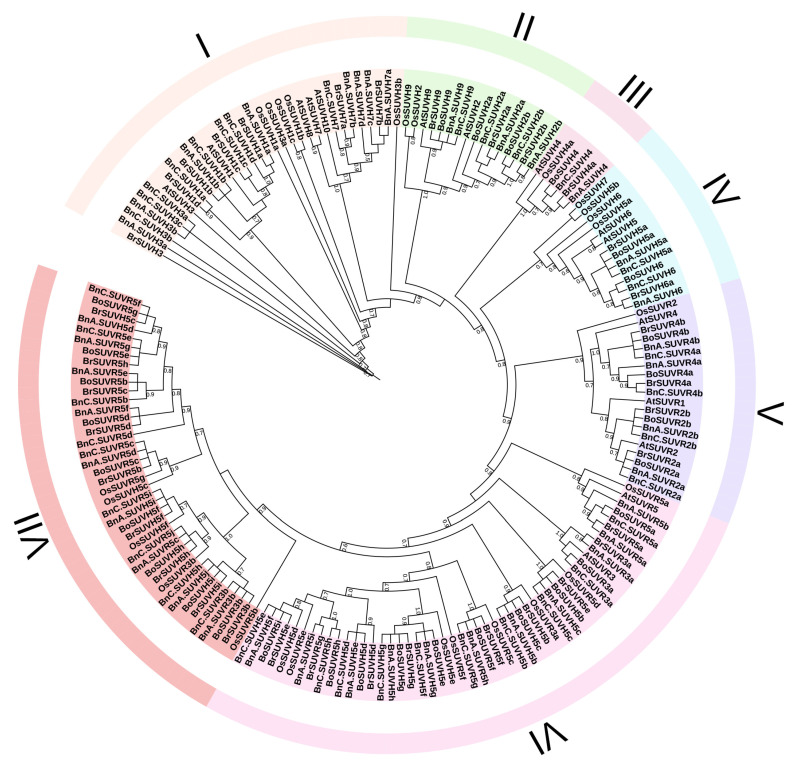
Phylogenetic tree of SUV gene family in *B. rapa*, *B. oleracea* and *B. napus*, Arabidopsis and rice, including 180 SUV proteins, was divided into I-VII seven groups. The bootstrap value which was greater than 50% was displayed at the base of the branch.

The same identification methods were adopted to identify the SUV gene family in rice. Then the SUV gene families in Arabidopsis and rice were taken as outgroups to construct the phylogenetic tree. A research about Su (var) 3-9 SET genes in land plants divided the SUV gene family in Arabidopsis into seven groups, namely V-1 to V-7, and these genes in each group had different functions [8]. According to the SUV proteins distribution of Arabidopsis in the phylogenetic tree, we divided the identified SUV proteins in *B. rapa*, *B. oleracea*, *B. napus* and rice which were homologous to Arabidopsis into the same group, and then combined with the actual clade situation of the phylogenetic tree, a total of seven groups were obtained (Figure 1). Among them, group III has only 6 members, while group VI has the largest number with 50 proteins. The members of the group I are SUVH1, SUVH3, SUVH7, SUVH8 and SUVH10 and members of group II are SUVH2 and SUVH9. The III group is the proteins coded by homologous genes of SUVH4, and the group IV contains SUVH5 and SUVH6. The group V is homologous to SUVR1, SUVR2 and SUVR4. These five groups are consistent with the previous study [8]. According to the real clade situation, we got the VII group and divided the remaining members into one group (The members were AtSUVR3, AtSUVR5, and the proteins encoded by their homologous genes in *B. rapa*, *B. oleracea*, *B. napus* and rice). In Arabidopsis, group I plays a major role in heterochromatic silencing, while group II plays a minor role in heterochromatic silencing [20,55]. Group IV can be used as a component of dimethytransferase [56]. From the member composition of groups VI and VII, SUVR3, SUVH5 and SUVR5 may be evolution-related, but their functions are still unknown.

More than 50% of the bootstrap value is displayed at the base of the branch. The larger the bootstrap value is, the higher the reliability of the branch is. Arabidopsis and rice homologous proteins were distributed on each group. Except for group VII, the AtSUV proteins distributed on each group were corresponding to the proteins encoded by orthologous genes in *B. rapa*, *B. oleracea* and *B. napus*, respectively, such as: AtSUVH2 and AtSUVH9 in group II were corresponding to BnSUVH2, BnSUVH9; BrSUVH2, BrSUVH9; and BoSUVH2, BoSUVH9, indicating that SUV proteins in *B. rapa*, *B. oleracea* and *B. napus* had highly homologous relationships with that in Arabidopsis. However, the SUV proteins in rice were not completely following this rule. For example, OsSUVH7 belongs to group IV which was different from the group IV classification of the SUV gene family in *Brassica* and Arabidopsis.

### 3.3. Gene Structure and Protein Conserved Domain

**Figure 2:**
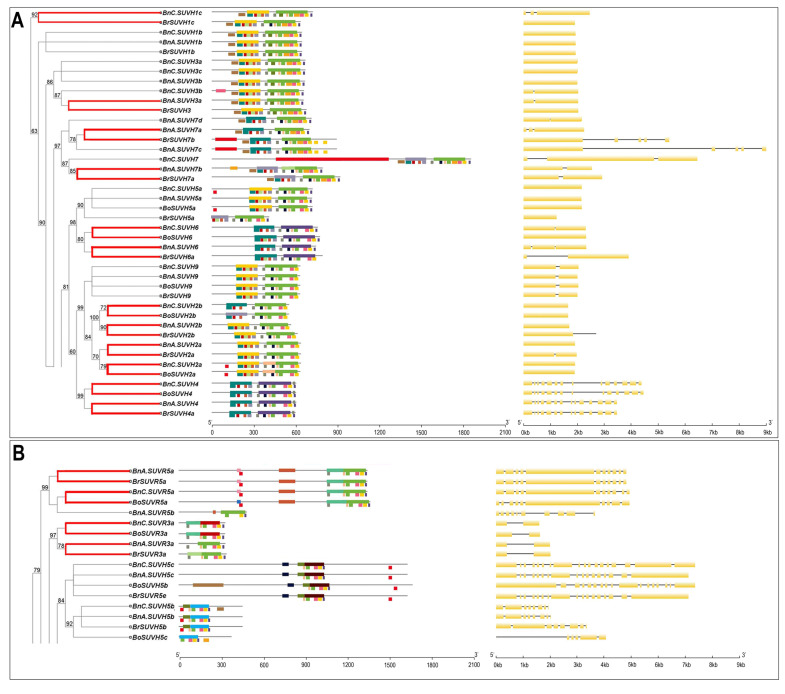

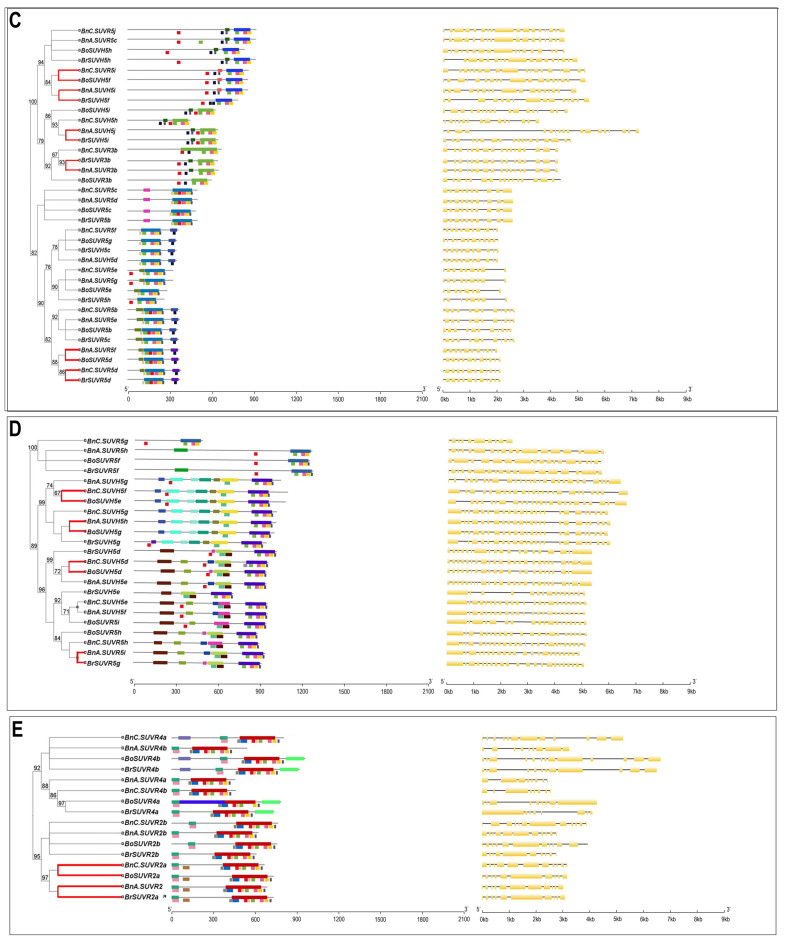

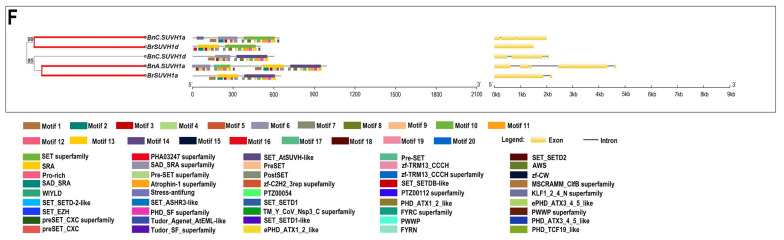
Intron/exon structure and conserved domain and motif overlap characterizations of the SUV gene family in *B. napus*, *B. rapa* and *B. oleracea*. (**A**–**F**): The characterizations of the SUV in the clade A to F. Note: The left part is the phylogenetic tree of the SUV gene family. In the red branches, 30 groups of orthologous gene pairs between *B. napus* and its diploid ancestors may have direct evolutionary relationships. The middle part is the overlap of protein domains and motifs (for each gene, the domain on the line and the motif under the line). The right part shows the gene structure.

To further analyze the conservation and evolution of the SUV gene family in *Brassica*, protein motif and gene structure were analyzed, and the specific results were shown in Figure 2A–F. According to the phylogenetic tree and the motif situation, six groups (A–F) were obtained. As can be seen from the figure, the number of introns in different branches varies from 0 to 25 (Table S1). Group A and group F have the smallest range of introns (from 0 to 4), while group D has the largest range of introns (from 9 to 25). As for orthologous genes of Arabidopsis and *Brassica*, the introns number of SUVR2 varies from 9 to 11, and SUVR3, SUVR4 and SUVR5 vary from 1 to 16, 7 to 12 and 7 to 25, respectively. Except for SUVH5, which has a large variation range of 0 to 25, the variation range of other SUVH genes is relatively small. SUVH4 and SUVH9 genes in *Brassica* have the same intron number, with 13 and 1, respectively. Both SUVH2 and SUVH3 vary between 0 to 1. The introns of SUVH1, SUVH6 and SUVH7 vary between 0 to 4, 0 to 2 and 1 to 4, respectively.

The branches of the phylogenetic tree in red indicated 30 orthologous gene pairs that may be evolution-related between *B. napus* and its diploid ancestors. We analyzed the gene structure of the 30 orthologous gene pairs and found that 12 (40%) orthologous gene pairs had an equal number of introns severally, which indicated that most of the orthologous gene pairs were consistent with the intron pattern. The numbers of introns in *B. napus* were amplified in 13 orthologous gene pairs. However, the introns in the remaining 5 orthologous genes pairs of *B. napus* were smaller than those in the diploid ancestors.

**Figure 3:**
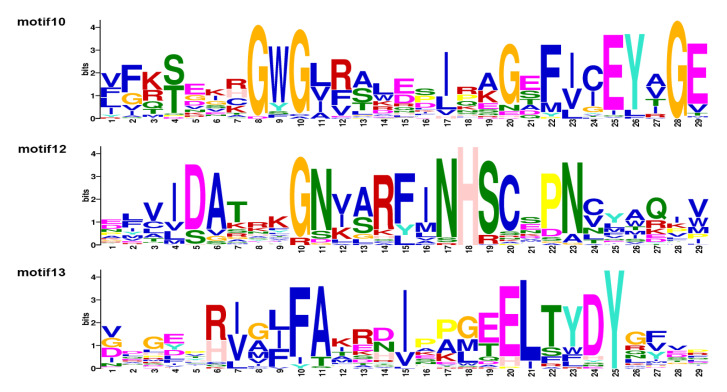
The sequence logos of conserved motifs in SUV gene family of *B. rapa*, *B. oleracea* and *B. napus*.

NCBI and MEME databases were used to analyze the domain and motif distribution. The results showed that all SUV genes contained the SET domain and other domains play a complementary role in each group. Groups A and F are conservative in SRA and SET domains. The difference is that most members in A contain other domains, such as Post-SET and Pre-SET. The SET domain is conserved in B, C and D, but they have different complementary domains: group B contains AWS, Pre-STE and ZF-CW, etc; group C contains CXC, PHD and AWS, etc; group D contains PHD, PWWP, FYRC and FYRN. In group E, the conserved domain model is WYILD-SET, and individual members contain stress-antifung domain. The motif analysis showed that all SUV proteins contained motif10 and motif13, and all SUV proteins except BoSUVR4a contained motif12, indicating that motif10, motif12 and motif13 were highly conserved among all SUV proteins in *Brassica*. As can be seen from the results shown in the figure, members in groups C and D all contain motif10, motif12 and motif13. The difference is that most proteins in group C contain motif9, 14, 15 and 16, while most proteins in group D contain motif14 and 17. Except for motifs10, 12 and 13, motif2, 3, 5, 7 and 9 are conserved in group A; motif14 is conserved in group B; motifs7, 9, 14, 16, 19 and 20 are conserved in group E; motif3, 5, 6, 7, 8 and 9 and 11 are conserved in group F. As can be seen from the overlaps of conserved domains and motifs, motif10, motif12 and motif13 (Figure 3) distributed in the SET domain, and conserved domains and conserved motifs also overlap each other in each group. Therefore, the SUV gene family of *B. napus* and its diploid ancestors could be divided into different groups according to the distribution of domains and motifs. 

The motifs analysis of 30 orthologous gene pairs found that 22 orthologous gene pairs had the same motif patterns and types, which indicated that most SUV proteins in *B. napus* were conserved in motifs during the polyploidization process.

### 3.4. Chromosomal Localization of SUV Genes

**Figure 4:**
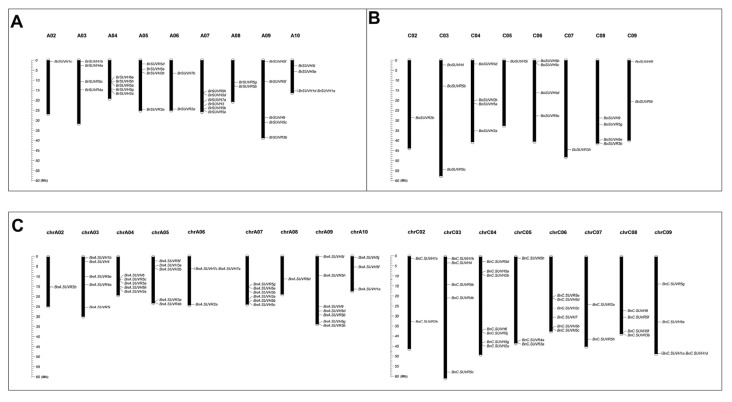
The chromosomal location of the SUV gene family in *B. rapa* (**A**), *B. oleracea* (**B**) and *B. napus* (**C**).

The chromosome physical locations of the SUV genes in *B. rapa*, *B. oleracea* and *B. napus* were analyzed (Figure 4). It was found that, except *BrSUVR2b* and *BrSUVR4b*, the other 33 SUV genes were accurately located on 9 chromosomes in *B. rapa*, but there was no SUV gene located on A01. In *B. oleracea*, 20 SUV genes were accurately located on 8 of 9 chromosomes, and 9 SUV genes in *B. oleracea* (*BoSUVR2a*, *BoSUVR3a*, *BoSUVR4a*, *BoSUVR4b*, *BoSUVR5a*, *BoSUVR5f*, *BoSUVH5g*, *BoSUVH5h* and *BoSUVH6*) were located on scaffolds. As for *B. napus*, a total of 36 genes were accurately located on 9 chromosomes, among which, chrA01 did not identify related genes, and two genes (*BnA.SUVH7b* and *BnA.SUVH7d*) were located on A_n_ subgenome. In the C_n_ subgenome, a total of 33 genes were located on 8 chromosomes, while chrC01 did not identify related genes, and 4 genes (*BnC.SUVR5a*, *BnC.SUVR5i*, *BnC.SUVH3a* and *BnC.SUVH3b*) were located in the C_n_ subgenome without precise location information (Table S1).

There were significant differences in the number of SUV genes located on different chromosomes. For example, 7 genes were located on chrA09, while only 1 SUV gene was located on chrA02 and chr08, respectively. Seven genes were identified on chrC04, while two SUV genes were identified on chrC02 and chrC07. In *B. rapa*, 6 genes were identified on A07, while only 1 gene was identified on A02. The distributions of SUV genes in *B. oleracea* were slightly different. Four genes were located in C04, C06 and C08 respectively, while only one SUV gene was located in C02, C05 and C07 respectively.

The results showed that *BrSUVH1a* and *BrSUVH1d* was a tandem gene cluster on A10 in *B. rapa*. There were two tandem gene clusters on chrA06 and chrC09 in *B. napus*, namely *BnA.SUVH7a* and *BnA.SUVH7c*, *BnC.SUVH1a* and *BnC.SUVH1d*. 75 BnSUV genes were identified in *B. napus*, of which 36 genes were accurately mapped on the A_n_ subgenome and 33 on the C_n_ subgenome, with little difference in number. Comparing the relative positions of these genes which were located on A_r_ and A_n_, C_o_ and C_n_ subgenome, it was found that for the relative positions of 26 SUV genes in *B. rapa* there were no changes in *B. napus*, and only 9 genes in *B. oleracea* kept the relative positions in *B. napus*.

### 3.5. Syntenic and Duplicated Gene Analysis

Syntenic genes can be used to describe the relationships between gene segments in different species, and the analysis of syntenic genes is critical to understanding the evolutionary relationships between different species from the same ancestor [57]. There were 18 SUV syntenic genes in *B. rapa*, 6 SUV syntenic genes in *B. oleracea*, 32 SUV syntenic genes in *B. napus* searched in the BRAD database (Table S3). However, the syntenic genes of *AtSUVR1*, *AtSUVH8*, *AtSUVH9* and *AtSUVH10* had not been searched in *Brassica*. The orthologous genes which were located in the same conserved block respectively distributed in 9 conserved blocks, namely A, E, F, G, I, J, R, T and V. The triplicated blocks of *Brassica* were divided into three subgenomes: LF, MF1 and MF2 [31]. The distribution quantity of SUV syntenic genes in different species differed in each subgenome. In *B. rapa*, 8, 8 and 2 genes distributed in LF, MF1 and MF2 subgenomes, respectively. There were 2 genes in LF and 4 genes in MF1 subgenome in *B. oleracea*, but no syntenic genes were found in MF2. The numbers of genes in LF, MF1 and MF2 subgenomes in *B. napus* were 13, 14 and 5. Most SUV genes (26 genes) distribute in the MF1 subgenome, while the least SUV genes (7 genes) distribute in the MF2 subgenome. In summary, a total of 56 SUV genes were searched in the syntenic region, accounting for 40% of the identified SUV genes, which was far less than the identified SUV genes, indicating that a large number of SUV genes in *B. napus* and its diploid ancestors were lost in the evolutionary process.

**Figure 5:**
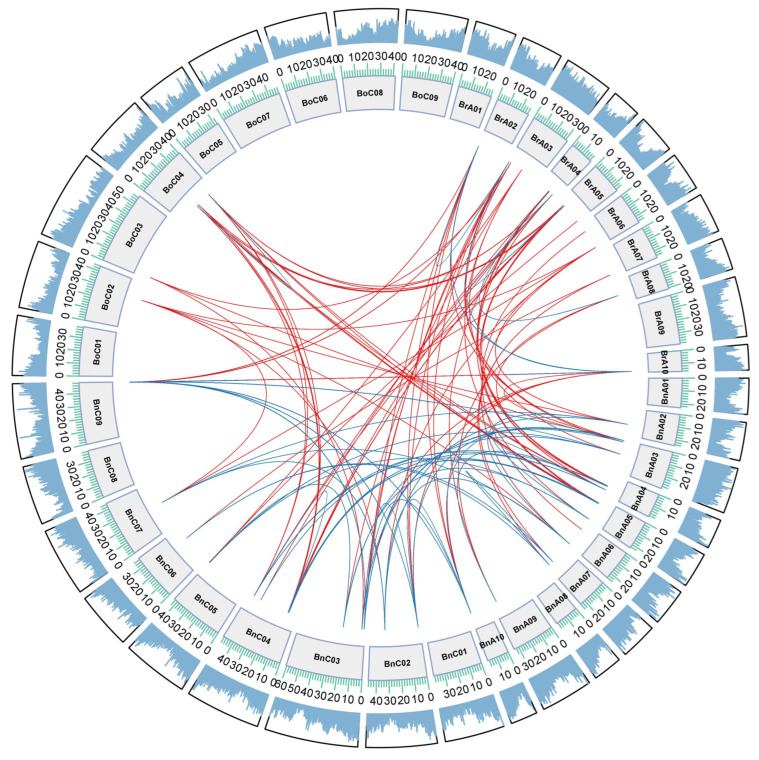
Genome-wide synteny analysis of A_n_ and C_n_ subgenome in *B. napus*, and A_r_ genome in *B. rapa*, and C_o_ genome in *B. oleracea*. Blue lines in the figure represented the paralogous genes and red represented orthologous genes. The outer ring was gene density on chromosome. The inner circle was the chromosome name and its length scale.

To more intuitively show the evolutionary relationships of syntenic genes between the SUV genes of *B. rapa*, *B. oleracea* and *B. napus*, the Circos was plotted as below (Figure 5). The corresponding syntenic genes of *AtSUVH4*, *AtSUVH5* and *AtSUVH6* identified in *B. rapa* and *B. oleracea* were retained in the same subgenome in *B. napus*. In addition, copies of the *AtSUVH1* gene in *B. rapa* were intact in all three subgenomes (LF, MF1, MF2) and kept well homologous to *BnA.SUVH1*, which would be related to the specific role of this gene during evolution. According to the syntenic relationship, two gene clusters, namely *BrSUVH1d* and *BrSUVH1a*, *BnC.SUVH1a* and *BnC.SUVH1d* were arranged in tandem, which was consistent with their chromosomal location information.

Tandem duplication and segmental duplication patterns of the identified SUV genes were analyzed. In *B. rapa*, *BrSUVH1a* and *BrSUVH1d* was a tandem gene cluster. There were two tandem gene clusters in *B. napus*, namely *BnC.SUVH1a*, *BnC.SUVH1d*, and *BnA.SUVH7a*, *BnA.SUVH7c*. No tandem gene cluster was identified in *B. oleracea*. It could be seen that the tandem gene cluster *BrSUVH1a* and *BrSUVH1d* in *B. rapa* were retained during the process of allopolyploidization in *B. napus*, but the *BnC.SUVH1a* and *BnC.SUVH1d* gene cluster was located on the C_n_ subgenome. Analysis of all the identified SUV genes showed that a total of 46 pairs of genes were segmental duplication, including 44 pairs of *B. napus* and 2 pairs of *B. rapa*. Similarly, no segmental duplicated genes were identified in *B. oleracea*. From both duplicated models, the number of gene pairs caused by segmental duplication was far more than that by tandem duplication, which suggested that segmental duplication played a more important role in the expansion of the entire SUV gene family in *B. rapa*, *B. oleracea* and *B. napus*.

Ka and Ks values of these segmental gene pairs were calculated to analyze the evolutionary pattern (Table S4). The results showed that the Ka/Ks values of 44 segmental gene pairs (about 95.7%) were less than 1.0, which were affected by the purifying selection. However, the Ka/Ks values of *BnA.SUVH2b*, *BnC. SUVH2b*, *BnA.SUVR5* and *BnC.SUVR5b* were greater than 1.0 and affected by the positive selection. In addition, the Ka/Ks values of 5 segmental gene pairs were found to be less than 0.1, indicating that they were strongly purified and selected.

### 3.6. The Gene Duplication Types and Transposable Elements Analysis

**Figure 6:**
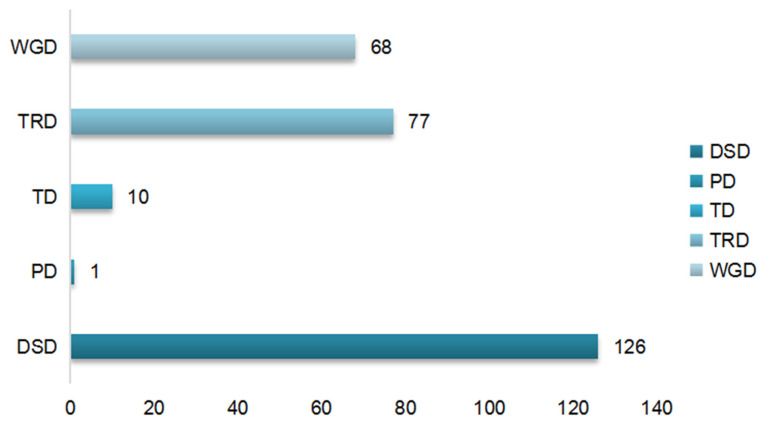
The duplication types of SUV gene family in *B. napus*, *B. rapa* and *B. oleracea*.

**Figure 7:**
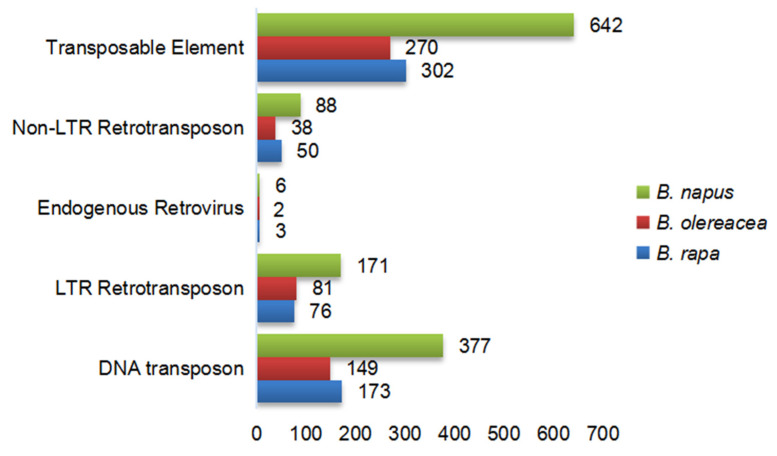
The number of TEs around 2000 bp upstream and downstream of SUV genes in *B. rapa*, *B. oleracea* and *B. napus*.

The gene duplication types can be classified as whole-genome duplication (WGD), Tandem duplication (TD), proximal duplication (PD), transposed duplication (TRD) and dispersed duplication (DSD) [58]. WGD is caused by the duplication of the whole genome. The mechanism of DSD is not clear. TD, PD and TRD are generated by single gene duplication. These duplication patterns were statistically analyzed to explore the reason for the SUV gene family duplication types in *B. napus* and its diploid ancestors (Figure 6). Results indicated that WGD, TRD and DSD appeared in most SUV genes, and the TD and PD were less than the previous three duplication types. The critical role of TRD in gene replication cannot be ignored, then 2000 bp upstream and downstream sequences around SUV genes were extracted to predict the transposable elements (Figure 7). There were 302, 270 and 642 transposable elements predicted around the SUV gene locus in *B. rapa*, *B. oleracea* and *B. napus*, respectively. DNA transposon, LTR Retrotransposon and Non-LTR Retrotransposon are the most TEs types in *B. napus* and its diploid ancestors. In conclusion, we found that the proportion of SUV genes with TRD was significantly higher than that of other three duplication types, and the number of TEs predicted near the SUV genes in *B. napus* was significantly more than two diploid ancestors, suggesting that the TRD which was mediated by TEs might be of great significance to the expansion of SUV gene family in *B. napus*.

### 3.7. Cis-Acting Elements in the Promoter Region of SUV Gene Family

**Figure 8:**
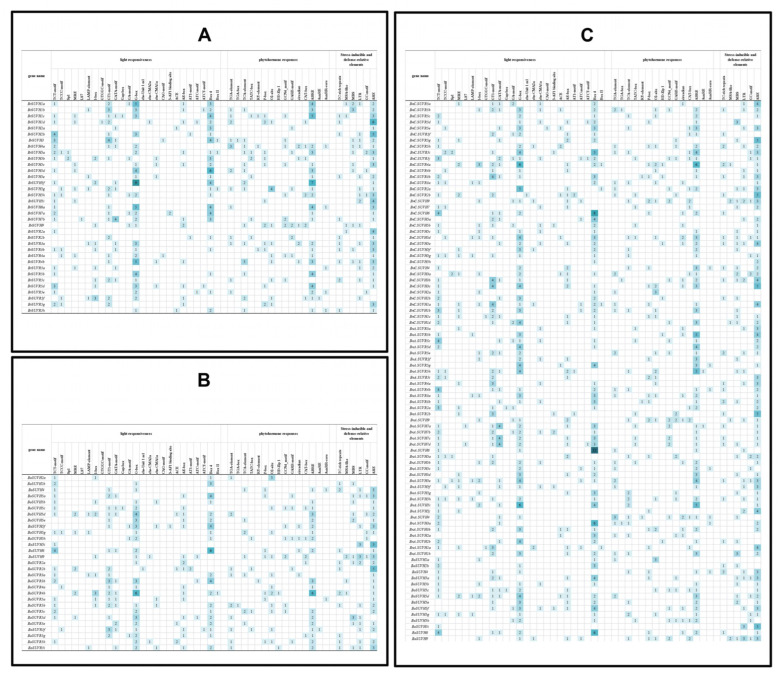
Number of elements responsive to stresses and hormones in the promoter regions of BrSUV (**A**), BoSUV (**B**) and BnSUV (**C**) genes.

In this study, 1500 bp sequence upstream of the gene transcription start site was extracted to *cis*-acting element analysis (Figure 8). There are 25 light-responsive elements, 15 phytohormone responsive elements, 6 stress-inducible and defense-relative elements. Phytohormone responsive elements and stress-inducible and defense-relative elements are crucial for plant defense regulation. In *B. napus*, light response elements varied between 1 to 12, phytohormone response elements varied between 1 to 6, and stress-inducible and defense-relative varied between 1 to 5. The SUV genes in *B. rapa* did not contain chs-Unit1 m1 and HD-Zip1 response elements, and the light-responsive elements, phytohormone response elements, stress-inducible and defense-relative elements varied from 1 to 9, 1 to 7, 1 to 6, respectively. The number ranges of *cis*-acting elements in *B. oleracea* are as follows: the light-responsive elements and phytohormone-responsive elements varied from 1 to 6, and the number of environmental stress elements varied from 1 to 5. Compared with *cis*-acting elements in *B. napus* and *B. rapa*, the number and types of *cis*-acting elements in *B. oleracea* were the least. All SUV genes in *B.* *oleracea* did not contain SP1, LS7, GTGGC-motif and CAG-motif. Overall, G-box, Box4, ABRE and ARE were significantly more than any other elements in *B. napus* and its diploid ancestors, which indicated that these four kinds of *cis*-acting elements were more conservative in *Brassica* transcription regulation. As for the 75 SUV genes in *B. napus*, 61 genes contained anaerobic induced element (ARE), 52 genes contained abscisic acid response element (ABRE), 36 genes contained GT1-motif element, 26 genes contained drought response element (MBS), 23 genes contained defense and stress response element (TC-rich repeats) and 21 genes contained low-temperature response element (LTR). These results suggest that the SUV genes in *B. napus* contain many environmental stress elements, which play an important role in the process of stress resistance.

From the previous analysis, there are 30 orthologous gene pairs which may have an evolutionary relationship. By analyzing the *cis*-acting elements of these 30 orthologous gene pairs, it was found that the types of *cis*-acting elements in *B. napus* were more than that of two diploid parent ancestors. Four orthologous gene pairs, namely *BnC.SUVH2a* and *BoSUVH2a*, *BnA.SUVH5i* and *BrSUVH5f*, *BnA.SUVH5j* and *BrSUVH5i*, *BnA.SUVH5i* and *BrSUVR5g* had the same CAREs types (And three orthologous gene pairs of them had equal *cis*-elements number). The above analysis results show that in the process of allopolyploidization, only some SUV genes are conserved in *cis*-elements, and the types and quantities of CAREs in *B. napus* are richer than those in *B. rapa* and *B. oleracea*, which indicates that the expression regulation in *B. napus* is more complex.

### 3.8. Expression Patterns of SUV Gene Family in Different Tissues

**Figure 9:**
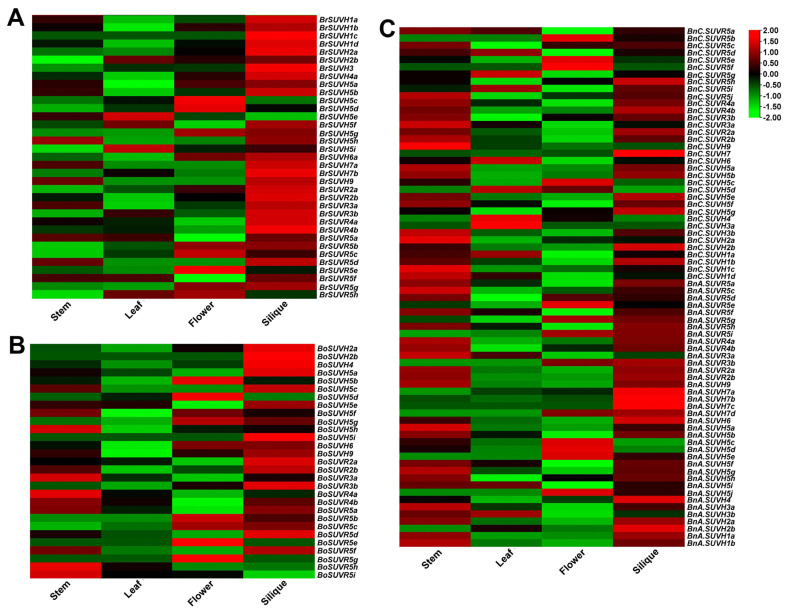
Expression patterns of SUV genes in four tissues (stem, leaf, flower, silique) of *B.* *rapa* (**A**), *B. oleracea* (**B**) and *B. napus* (**C**).

Combining with the RNA-seq data of our research group [37], the SUV gene expression levels of *B. rapa* (A), *B. oleracea* (B) and *B. napus* (C) were plotted into the heat map as shown in Figure 9 (The darker the red, the higher the expression, and the darker the green, the lower the expression). Three SUV genes (*BnC.SUVH5h*, *BnC.SUVH3c* and *BnA.SUVR5b*) did not find expression data in four tissues. It was found that most SUV genes in *B. rapa* and *B. oleracea* showed high relative expression levels in siliques (Table S5). In *B. napus*, *BnC.SUVR5e*, *BnC.SUVR5f* and *BnA.SUVR5e* were specifically highly expressed in flowers. Compared with the other three tissues, *BnC.SUVH4* and *BnC.SUVH3a* had the greatest expression levels in leaves. The expression levels of *BnC.SUVH2a* and *BnC.SUVH9* were the highest in stems. Four SUV genes in *B. napus*, namely *BnA.SUVH7a*, *BnA.SUVH7b*, *BnA.SUVH7c* and *BnC.SUVH7* were specifically highly expressed in siliques. In addition, the expression levels of 27 BnSUV genes were higher in stems and siliques than in flowers and leaves. *BnC.SUVR5i* and *BnA.SUVH2b* were highly expressed in leaves and siliques. The expression level of *BnC.SUVH5d* in leaves and flowers was higher than that in stems and siliques. *BnA.SUVR5g*, *BnA.SUVR5i*, *BnA.SUVR3b*, *BnA.SUVH7d*, *BnA.SUVH5e*, *BnA.SUVH5j* and *BnC.SUVR5b* were highly expressed in flowers and siliques. *BnA.SUVR3a* and *BnA.SUVH3b* were highly expressed in stems and leaves. The expression levels of three genes *BnA.SUVH5c*, *BnA.SUVH5d* and *BnC.SUVH5c* in stems and flowers were higher than those in leaves and siliques. In conclusion, most of the SUV genes in *B. napus* are highly expressed in stem and siliques, and different BnSUV genes show different tissue expression patterns.

Based on the previous analysis, 30 orthologous gene pairs which may have evolutionary relationships were obtained. The expression patterns of these orthologous genes were analyzed, and it was found that the expression patterns in these four tissues of most BnSUV genes were different from the SUV genes in diploid ancestors. Among them, *BnA.SUVH1a* and *BrSUVH1a*, *BnA.SUVH4* and *BrSUVR4a*, *BnC.SUVR2a* and *BoSUVR2a* had the same expression patterns and were expressed highly in stems and siliques. The expression levels of *BnA.SUVR5i* and *BrSUVR5g* were higher in flowers and siliques. In addition, the relative expression levels of most BnA.SUV, BrSUV and BoSUV genes in siliques were higher than those in the other three tissues, while the relative expression levels of most BnC.SUV genes in leaves were higher than those in the other three tissues.

To understand the subgenomic expression bias of the 30 orthologous gene pairs, we divided them into 10 groups. Among them, 2 groups only appeared in one ancestor, and 2 groups did not correspond to the corresponding subgenomic SUVR or SUVH genes. The remaining 6 groups, namely SUVH2, SUVH5, SUVH6, SUVR2, SUVR3 and SUVR5 were analyzed for subgenomic bias as shown in Table 1.

The expression of SUVH5 in all four tissues was biased to the C_n_ subgenome (*B. oleracea*), while the expression of SUVR3 and SUVR5 in all four tissues was biased to the A_n_ subgenome (*B. rapa*). The expression of SUVH2 in leaves was biased to the A_n_ subgenome, while that in the other three tissues was biased to the C_n_ subgenome. The expression of SUVH6 in leaves was biased to the A_n_ subgenome. SUVR2 was biased C_n_ subgenomes in stems and flowers, and A_n_ subgenomes bias in leaves and siliques. The differences in subgenomic expression bias of these genes in different tissues may be related to the functional differentiation of SUV genes and play a significant role in plant response to various environmental stresses.

## 4. Discussion

With the further study of the SET gene family, the role of the SUV gene family in methylation has been gradually revealed. Except for the Pre-SET, SET and Post-SET domains, two categories (SUVH and SUVR) of the SUV gene family in Arabidopsis could be obtained by the distribution of two domains, namely SRA and WIYLD [3,4,5,13,29]. So far, most research on the SUV gene family is related to the SET gene family [15,16,17,18,19]. Only the SUV gene family in *B. rapa* has been reported, but the SUV gene families in *B. oleracea* and *B. napus* are still blank. In this study, we analyzed the SUV gene family in *B. napus* and its diploid ancestors and revealed the changes of the SUV gene family during *Brassica* evolution and allopolyploidization.

### 4.1. Compared with Its Diploid Ancestors, SUV Gene Family in B. napus Amplified during Allopolyploidization

The SUV gene members identified in *B. napus* was significantly more than the sum of the SUV genes in the two diploid ancestors, indicating that the SUV gene family in *B. napus* was amplified during the process of allopolyploidization. In our study, three tandem gene clusters and 46 pairs of segmental genes were identified, suggesting that segmental genes play a critical role in the expansion of the SUV gene family in *B. napus*. The mechanisms of gene duplication can be divided into two forms: whole-genome doubling and single gene duplication, namely WGD, TD, PD, TRD and DSD [58]. The TRD was significant to the SUV gene duplication and the analysis of TEs indicated that there were 302, 270 and 642 transposable elements in *B. rapa*, *B. oleracea* and *B. napus*. The fates of homologous genes can be divided into silencing, neofunctionalization and subfunctionalization [59]. The duplicated genes produced by different mechanisms had significant structural differences. Among them, the duplicated genes produced by transposable duplication had the greatest structural differences, and such structural differences were related to their expression differences [60]. The retention mechanism of duplicated genes is positively correlated with its function. The gene function, the number of interacting proteins and the gene structure may affect the fate of the duplicated genes, and the origin of pseudogenes also has a certain functional bias [61]. In addition, the mechanism and function of duplicated genes not only affect the fate differentiation of genes but also affect the lifespan of duplicated genes [62].

### 4.2. Some Orthologous Genes of SUV Gene Family in Arabidopsis Were Lost in B. napus and Its Diploid Ancestors during Evolution

As a common source of genetic variation and one of the most important evolutionary forces in biology, gene loss which is mainly caused by physical removal and pseudogenization mechanisms shows obvious bias in gene function and genomic position and is affected by the dosage balance [63]. Whole-genome duplication event (WGD) is one of the important forces driving plant evolution [64]. Before the separation of *Brassica* and Arabidopsis lineages, the WGD event was experienced, and then another whole-genome triploid (WGT) event was experienced [65,66]. After the WGT event, the gene family menbers changed, and the amplification phenomenon was also identified in the 2OGD gene family [67]. WGD is closely related to the evolution of plants and the origin of polyploidy and two hypotheses had been proposed to explain the origin and evolution of polyploidy [68]. Actually, WGD is not only one of the causes of gene family amplification but also affects the *Brassica* genome structure [69]. After the separation of *Brassica* and Arabidopsis lineages, the WGT event appeared, but in fact, the diploidization process resulted in the loss of *B. rapa* genome length and when WGT event occurred in *Brassica* lineages, 35% of the genes presumed to be present have been lost [32]. Due to genome shrinkage, the triploidization *B. rapa* genome contains only about twice as many genes as the Arabidopsis genome, of which fewer tandem duplicates in the *B. rapa* genome may be attributed to the increased deletion rate [70]. The size difference between *Brassica* A and C genomes appears to be largely due to transposable elements inserted throughout the genome after speciation. Allopolyploidy leads to chromosomal rearrangement, and chromosomal structural variation is common in both natural and synthetic allotetraploids [32,71]. In *B. napus*, some gene fragments were lost due to chromosomal rearrangement, and phenotypic variation was produced [72]. The pseudogenes (loss of gene function) are positively correlated with the scale of the gene family [60]. Some researchers reported that *SUVH7* and *SUVH10* were probably pseudogenes in Arabidopsis [2,7]. In this study, 56 SUV genes were identified through syntenic gene search, accounting for about 40% of the SUV genes in *B. napus* and its diploid ancestors, which was far less than the number (139) of SUV genes identified, indicating that a large number of SUV genes were lost in the evolutionary process. This may be related to the occurrence of polyploidization events and environmental factors during the evolutionary process of *Brassica*.

### 4.3. The Gene Structure of SUV Gene Family Was Conserved during the Allopolyploidization Process

According to the distribution of SRA and WIYLD domains, the SUV gene family was divided into SVUH and SUVR in Arabidopsis [4]. Evolutionary analysis of the SET gene family revealed that SRA and WYILD were found only in plants [7]. Except for the SET domain, the SUV gene family also contains several other domains that complement the function of SUV proteins. The Post-SET domain is critical for the formation of SAM-binding pockets which is necessary for methyltransferase catalytic activity [13]. The SRA domain plays an important role in DNA methylation and works with the SET domain to identify cytosine methylation sites in DNA which is closely related to H3K9 methylation modification [22,73]. The WIYLD domain originated from marchantiophyta [2], and related studies have shown that the WIYILD domain can bind ubiquitin, thereby affecting the methylation of histone [28]. From the evolutionary relationship, SAR, ZnF_C2H2 and WIYLD domains are integrated into PreSET/SET/PostSET at an early stage, to promote differentiation [8]. In this study, the SUV gene family of *B. napus* and its diploid ancestors was divided into six groups (A–F) by the reference criteria of the domain and motif distribution. The result showed that except for SRA, SET and WIYLD domains, each group also contained different domains, such as PHD, AWS, Pre-SET-CXC, Pre-SET and Post-SET domains which were different from the structure distribution of the SUV gene family in Arabidopsis [2]. By analyzing the gene structure, domain and motif of 30 orthologous gene pairs, 12 pairs (40%) had the same number of introns, and 22 pairs (70%) had the same number of motifs and distribution patterns. These results suggest that the SUV gene family in *B. napus* is conserved at the DNA and protein levels during allopolyploidization.

### 4.4. The Expression Patterns of SUV Genes in B. napus Were Changed

Gene expression is regulated by a variety of mechanisms, such as methylation, which plays an important role in gene silencing [74], and the importance of miRNA in the post-transcriptional regulation of gene expression [75]. Based on whether gene expression is constitutive or induced, ubiquitous or cell-specific, there are four gene expression patterns, namely constitutive, signal-dependent, inducible, and cell-type specific [76]. In this study, the expression data of 30 orthologous gene pairs show different patterns, and only 4 (13%) orthologous gene pairs are the same in tissue expression patterns. There are 40% of orthologous gene pairs that maintained the same intron quantity, and about 70% of orthologous genes conserved at the protein level. This indicates that although most SUV orthologous genes are conserved at the DNA and protein levels, only a few show the same expression patterns. In addition, these three SUV genes, namely *BnC.SUVH5h*, *BnC.SUVH3c* and *BnA.SUVR5b* did not find expression data in four tissues which may have different spatial and temporal expression patterns. The subgenomic expression bias of SUVH2, SUVH5, SUVH6, SUVR2, SUVR3 and SUVR5 may be related to functional diversity and stress resistance.

## 5. Conclusions

In this study, 139 SUV genes in *B. napus* and its diploid ancestors were obtained and analyzed. We found that the SUV gene family in *B. napus* amplified during allopolyploidization, and the segmental duplication was more important than the tandem duplication for amplification. Compared with Arabidopsis, many SUV genes in *B. napus* and its diploid ancestors were lost during evolution. Most SUV genes in *B. napus* are conversed in gene structure, but only 4 orthologous gene pairs had the same expression patterns. These results provide a reference for the study of the SUV gene family in polyploids and deepen our understanding of the SUV gene family in *Brassica*.

## Figures and Tables

**Table 1:** The |log_2_FC| of SUV genes in four tissues.

Gene Name	|log_2_FC| in Stems	|log_2_FC| in Leaves	|log_2_FC| in Flowers	|log_2_FC| in Siliques
SUVH2	0.83	2.42	0.42	0.64
SUVH5	0.71	0.17	0.48	0.33
SUVH6	2.33	0.72	1.45	1.53
SUVR2	0.69	2.45	0.43	1.29
SUVR3	3.70	3.32	2.61	2.36
SUVR5	2.46	1.07	2.98	2.31

Note: The value of |log_2_FC| > 1 means the gene expression was biased to *B. rapa*, otherwise, |log_2_FC| < 1 means it was biased to *B.* *oleracea*, and |log_2_FC| = 0 means this gene had no obvious bias.

## Data Availability

The raw data of RNA-seq reads were deposited in the NCBI database under accession number (SRR7816633-SRR7816668).

## Supplementary Materials

The following are available online at https://www.mdpi.com/article/10.3390/genes12121848/s1, Table S1: The SUV gene family information in *B. napus* and its diploid ancestors, Table S2: The physicochemical parameters and subcellular localization prediction of SUV proteins in *B. napus* and its diploid ancestors, Table S3: Syntenic gene analysis of SUV genes in Arabidopsis, *B. rapa*, *B. oleracea* and *B. napus*, Table S4: Estimated Ka/Ks ratios of BnSUV, BrSUV duplication gene pairs, Table S5: The relative expression values of SUV gene family in *B. napus* and its diploid ancestors.
